# High-frequency ultrasound analysis of post-mitotic arrest cell death

**DOI:** 10.18632/oncoscience.301

**Published:** 2016-04-15

**Authors:** Maurice M. Pasternak, Lauren A. Wirtzfeld, Michael C. Kolios, Gregory J. Czarnota

**Affiliations:** ^1^ Department of Laboratory Medicine and Pathobiology, University of Toronto, Toronto, ON M5S 1A8, Canada; ^2^ Department of Physical Sciences, Sunnybrook Health Sciences Centre, Toronto, ON M4N 3M5, Canada; ^3^ Department of Physics, Ryerson University, Toronto, ON M5B 2K3, Canada; ^4^ Department of Radiation Oncology, Sunnybrook Health Sciences Centre, Toronto, ON M4N 3M5, Canada; ^5^ Departments of Medical Biophysics, and Radiation Oncology, Faculty of Medicine, University of Toronto, Toronto, ON M4N 3M5, Canada

**Keywords:** quantitative ultrasound, breast cancer, imaging, midband fit, spectral slope

## Abstract

Non-invasive monitoring of cancer cell death would permit rapid feedback on treatment response. One technique showing such promise is quantitative ultrasound. High-frequency ultrasound spectral radiofrequency analysis was used to study cell death in breast cancer cell samples. Quantitative ultrasound parameters, including attenuation, spectral slope, spectral 0-MHz-intercept, midband fit, and fitted parameters displayed significant changes with paclitaxel-induced cell death, corresponding to observations of morphological changes seen in histology and electron microscopy. In particular, a decrease in spectral slope from 0.24±0.07 dB/MHz to 0.04±0.09 dB/MHz occurred over 24 hours of treatment time and was identified as an ultrasound parameter capable of differentiating post-mitotic arrest cell death from classical apoptosis. The formation of condensed chromatin aggregates of 1 micron or greater in size increased the number of intracellular scatterers, consistent with a hypothesis that nuclear material is a primary source of ultrasound scattering in dying cells. It was demonstrated that the midband fit quantitatively correlated to cell death index, with a Pearson R-squared value of 0.99 at p<0.01. These results suggest that high-frequency ultrasound can not only qualitatively assess the degree of cancer cell death, but may be used to quantify the efficacy of chemotherapeutic treatments.

## INTRODUCTION

As the diversity of chemotherapeutic options for malignant tumour treatment increases, the detection of treatment response becomes imperative as cancers may start out as sensitive responders, only to develop therapeutic resistance after multiple rounds of chemotherapy. Notably, breast cancers are notorious for the development of chemotherapeutic resistance; possibly through alterations to essential gene products such as Bcl-2, p21, and p53 [1]. Presently, no clinical modality exists to non-invasively evaluate the efficacy of therapy in the short-term - within hours to a few days after drug administration.

Current clinical imaging techniques such as X-ray computed tomography and positron emission tomography techniques share weaknesses, including their use of ionizing radiation, relative expense, and associated technical issues resulting from the poor retention of contrast agents [2, 3]. In comparison, high frequency ultrasound (HFUS; 20-60 MHz) coupled with spectral quantitative ultrasound analyses offers a non-invasive, high-resolution, and cost-effective imaging approach. At central frequencies of 25 MHZ and 40 MHz, the ultrasound wavelengths are 60 μm and 37.5 μm, respectively. It has previously been demonstrated that spectral ultrasound is sensitive to changes in physical properties of tissues, including the scatterer number density, bulk modulus, and other factors. It is well documented that chemotherapeutically-induced tumour cell death is accompanied by vast structural changes leading to alterations in physical properties [4], and therefore ultrasound imaging over treatment could permit the monitoring of the treatment efficacy through the quantification of cell death [5, 6].

Spectral techniques are sensitive to changes in structure in the sub-wavelength range, [7] permitting sensitivity to the size of target cells and their nuclei (~20- 50 μm and ~2-8 μm in diameter, respectively) [8, 9, 10]. These changes are reflected in the spectral analysis, which provides frequency-dependent information relating to the acoustic and structural properties of sample tissue [11, 12, 13]. Within this study, estimated parameters studied with chemotherapy-induced cell death include frequency-dependent attenuation, speed of sound [14], spectral intercept, spectral slope, and midband fit [15]. Additionally, a fluid filled sphere model was fitted to the backscatter coefficient, a fundamental material property, to permit estimates of the effective acoustic scatterer diameter and concentration which can provide information on the sizes of objects scattering the ultrasound waves and the number density combined with the relative impedance change of these objects, respectively [16, 17].

Previous studies have demonstrated the sensitivity of high-frequency ultrasound to apoptosis and necrosis for *in vitro* and *in vivo* samples [18]. In those studies, the detection of apoptosis was marked by a substantial increase in the integrated backscatter intensity as well as increases in the spectral slope associated with therapeutically-induced programmed cell death. In addition, a study by Vlad *et al*. [5] suggests that high frequency ultrasound is capable of differentiating varying modes of cell death responses, since cell death by radiation-induced death post-mitotic arrest produced a different set of acoustic parameter changes, particularly the spectral slope, which decreased by 20-40% in samples treated with radiation. This is a crucial component to consider, as chemotherapeutic treatment may induce mechanisms of death that are distinct from the background level of tumour cell death that is often responsible for false-positive results in PET scans [19]. The discernment of the types of cell death may help to eliminate such false-positives and give support as to whether the administered chemotherapeutic is effective or not.

Whereas general trends in acoustic parameters with cell death have been reported, several of these studies have implemented longer times (≥48 hours) after treatment and none have yet established a well-defined quantitative relationship between the number of cells in the death programme and changes in acoustic parameters. In this study, it was investigated whether a correlation exists between the cell death index of a tumour-mimicking cancer cell population and acoustic parameters. In addition, we investigated whether the different changes in acoustic parameters resulting from radiation-induced death were recapitulated through a chemotherapy procedure reported to induce the same mode of cell death.

## RESULTS

### High frequency ultrasound power spectra and histology in the course of paclitaxel treatment

**Figure 1:**
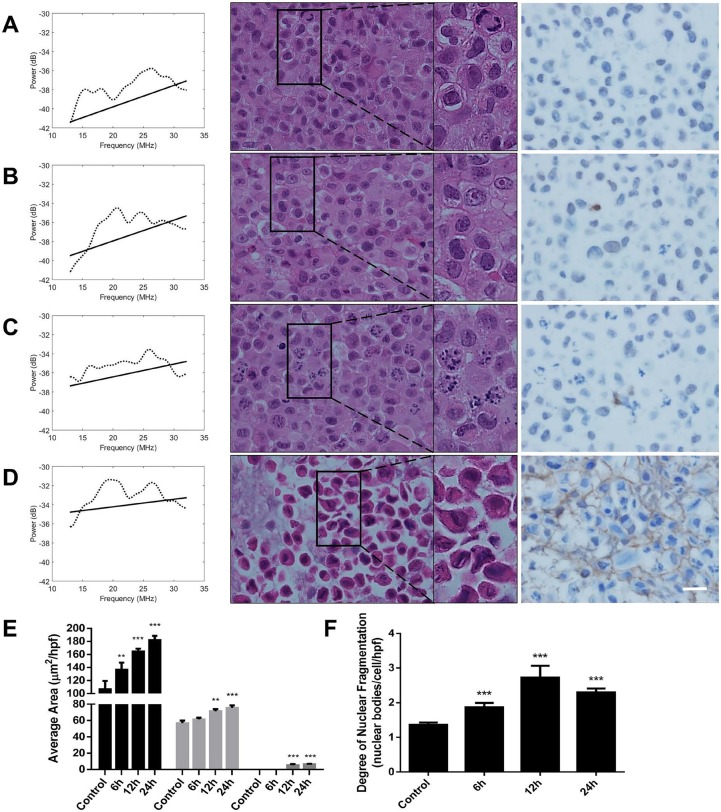
Normalized power spectra (left column), Haematoxylin and eosin stain (centre column), and ISEL & toluidine blue stain (right column) for A. Time-match control B. 6 hour paclitaxel, C. 12 hour paclitaxel, and D. 24 hour paclitaxel treatment exposures Black square regions display magnifications of cells of interest in the Haematoxylin and eosin panels. The scale bar represents 20 μm for all histology. Image analysis of histology of **E.** average area of cells and nuclei, and **F.** the number of nuclear bodies per cell for paclitaxel treatment. n≥3 for all conditions. * (p<0.05), ** (p<0.01), *** (p<0.001).

Normalized power spectra from paclitaxel-treated MDA-MB-231 cells (Figure 1) and colchicine-treated AML5 cells demonstrated increases in backscatter as well as decreases in spectral slope as a function of treatment time for both cell lines. The midband fit at 25 MHz increased from −37.2 dBr to −34.4 dBr whereas the spectral slope at 25 MHz decreased from 0.24 dBr/MHz to 0.12 dBr/MHz between time-matched control and 24 hours of paclitaxel exposure. The spectral intercept at 25 MHz also increased over this same comparison from −42.4 dBr to −36.5 dBr.

The timing of changes in power spectra strongly corresponded to changes in gross morphological alterations, as observed by haematoxylin and eosin staining as well as ISEL with toluidine blue counterstain. Initially, cells presented with a uniform staining of nuclear material (Figure 1A), followed by condensation and migration of chromatin to the periphery of the intact nucleus at 6-hours (Figure 1B). Following, the nuclear membrane appeared disassembled and highly-condensed collections of chromatin were visible in certain cells (Figure 1C). Further progression of cell death featured indicated staining of highly-condensed nucleic acid as well as ejection of ISEL-positive strands of nuclear material to the extracellular space at 24 hours (Figure 1D).

There were corresponding changes in the sizes of cells and the number of nuclear fragments, in which cellular cross-sectional area changed on average from 106.7 ± 12.7 μm^2^ to 182.1 ± 6.4 μm^2^ from control to 24 hours of treatment, respectively (Figure 1E). The number of nuclear fragments increased from 1.36 ± 0.07 nuclear bodies per cell body to 2.30 ± 0.11 nuclear fragments per cell body between control and 24 hours (Figure 1F).

**Figure 2:**
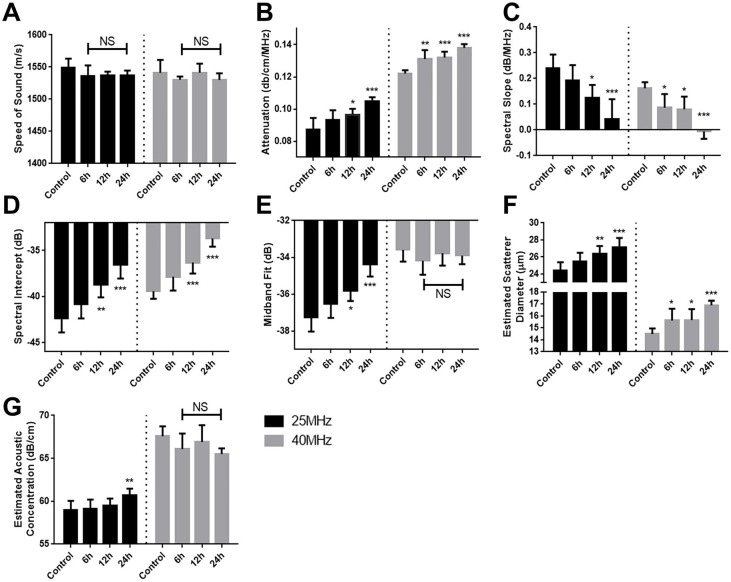
Changes in A. speed of sound, B. attenuation, C. spectral slope, D. spectral intercept, E. midband fit, F. effective acoustic scatterer diameter, and G. effective acoustic scatterer concentration ultrasonic parameters as a function of paclitaxel treatment duration All measurements were performed using two transducers with 25 and 40MHz centre frequencies. Error bars represent standard deviation. n=8 for all conditions. * (p<0.05), ** (p<0.01), *** (p<0.001).

RF Data was further analyzed to ascertain the values of quantitative ultrasound acoustic parameters. For all frequencies, the speed of sound (Figure 2A; Figure S1A) was not significantly different in the course of chemotherapeutic treatment, with a value of approximately 1540 m/s. Attenuation values (Figure 2B) increased as a function of treatment time at both 25 MHz and 40 MHz frequencies, with the latter detecting significant changes in attenuation at the earlier 6-hour time point. At 25 MHz, attenuation increased from 0.087 ± 0.008 dB/cm/MHz and 0.105 ±0.003 dB/cm/MHz between control and 24 hours.

It was observed that the spectral slope decreased with chemotherapeutic treatment time (Figure 2C; Figure S1B). Again, the higher 40 MHz frequency detected significant decrease as early as 6 hours. Spectral slope decreased from 0.24±0.07 dB/MHz to 0.04±0.09 dB/MHz and from 0.16±0.03 dB/MHz to −0.01±0.03 dB/MHz for 25 MHz and 40 MHz frequencies, respectively. Similar results were observed for the colchicine-treated AML5 cells, with an overall decrease from 0.33±0.05 dB/MHz to 0.23±0.05 dB/MHz.

In addition, the spectral intercept displayed increases at both frequencies as a function of treatment time for both MDA-MB-231 cells (Figure 2D) and AML5 (Figure S1C). Midband fit also demonstrated an increasing trend for MDA- 231 cells, but only at 25 MHz, with no significant changes observed throughout treatment at the 40 MHz central frequency (Figure 2E). For AML5 cells, the midband fit demonstrated a very significant increase from −56.6±0.7 dBr to −48.15±1.4 dBr over the course of 24 hours (Figure S1D).

The Fluid-Filled Sphere Model estimates of the effective scatterer diameter and effective acoustic concentration for MDA-MD-231 cells are summarized in Figure 2F and 2G, respectively. The effective scatterer diameter estimate increased throughout treatment. As with the midband fit, it had been observed that the 25 MHz frequency displayed more pronounced increases in estimated acoustic concentration.

### Paclitaxel induces a post-mitotic arrest form of death in MDA-MB-231 cells

**Figure 3:**
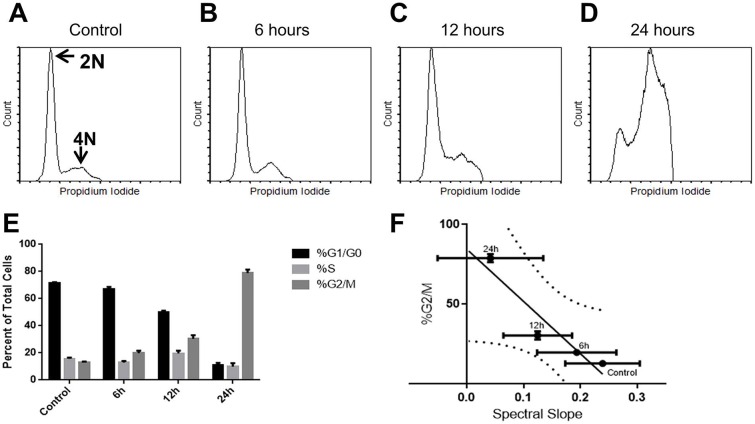
Flow cytometric analysis of DNA content as a function of treatment for A. no treatment (71.4%, G1/G0; 15.7%, S; 12.8%, G2/M), B. 6 hour (67.2%, G1/G0; 12.9%, S; 19.8%, G2/M), C. 12 hour (49.9%, G1/G0; 19.7%, S; 30.4%, G2/M), and D. 24 hour (11.2%, G1/G0; 10.0%, S; 78.9%, G2/M) paclitaxel The relatively high concentration of paclitaxel prevented complete mitotic division from occurring, indicated by the absence of a polyploid 8N or 16N populations. **E.** Graphical representation of cell phase percentages, indicating decreasing G1/G0 populations, relatively s-phase populations, and increasing G2/M populations. **F.** A linear correlation between the percent G2/M population and spectral slope at 25MHz from indicated time points and control. Curved lines indicate 95% confidence bands of regression lines. The goodness of fit was r^2^ = 0.865.

In order to correlate the spectral parameter trends to physical changes in the individual cells, flow cytometry was used to determine the predominant phase the cells were in at each time point. It was observed that the percentage of G1/G0 cells (2N peak) decreased whereas the percentage of G2/M cells (4N peak) increased between the time-matched control and 24-hour treatment. The percentage of S-phase cells remained approximately constant over time (Figure 3A-3E). This was consistent with the primary cell death modality occurring post-mitotic arrest, as cells will contain double the interphase content of DNA while dying in mitosis and not progressing to G1. Colchicine treatment of AML5 cells produced similar results (Figure S1E-S1F), with a substantial increase in G2/M phase cells from 8.7±2.5 % to 29.7±6.1 % by 24 hours of treatment.

The percentage of G2/M phase cells was plotted against the quantitative ultrasound parameters, with the best correspondence with spectral slope parameter. The strongest correlation resulted from the comparison of 25MHz spectral slope and the percentage G2/M cells, with a Pearson coefficient of −0.945 (*p*<0.05) (Figure 3F). A regression fit of r^2^=0.894 suggested that spectral slope may be used as a surrogate marker to ascertain increases in the number of cells within G2/M phase as a result of mitoticarresting chemotherapeutic treatment.

**Figure 4:**
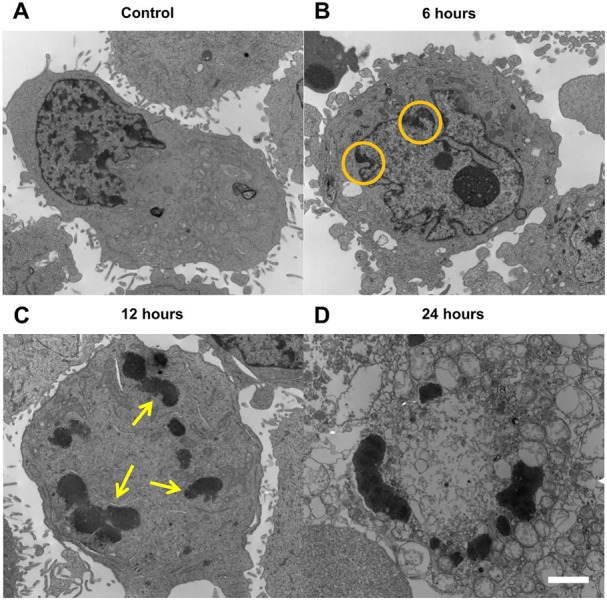
Transmission electron microscopy of MDA-MB-231 cells **A.** Control sample featuring normal cell morphology, with an intact nucleus containing relatively dispersed chromatin and an intact organelle network. **B.** After 6 hour paclitaxel treatment, pockets of condensed chromatin (micro-blebs, indicated within orange circles) appear in areas of the intact nuclear envelope. **C.** At 12 hour paclitaxel treatment, the nuclear envelope is compromised, with the formation of highly condensed blobs of nuclear material (indicated by yellow arrows) in a manner characteristic of mitotic catastrophe. **D.** At 24 hour paclitaxel treatment, the condensed chromatin blobs remain and extreme vacuole formation takes place without incorporating the condensed nuclear material. Scale bar indicates 2μm.

Using transmission electron microscopy (Figure 4), highly-condensed chromatin aggregates were observed at the 12-hour time. Cells undergoing post-mitotic arrest cell death were also TUNEL-negative (Figure S2) rendered positive only with DNAse treatment.

### High-frequency ultrasound midband fit correlates with cell death index

**Figure 5:**
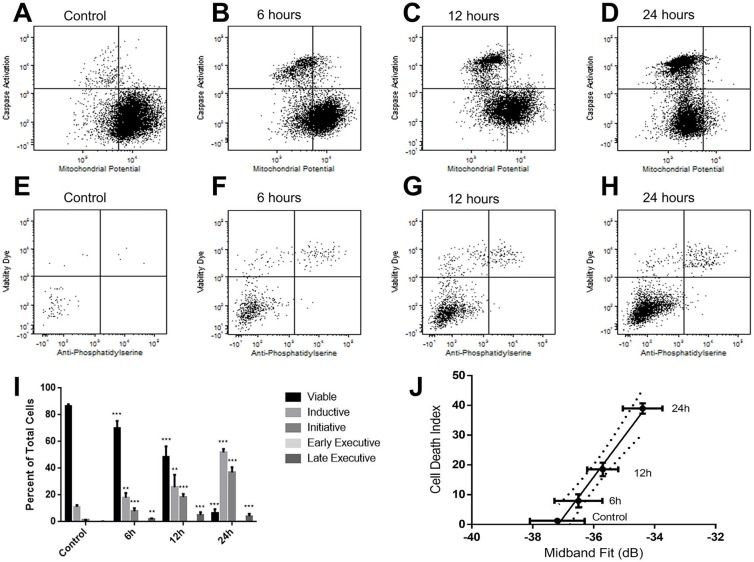
Flow cytometric analysis of cell death stages as a function of paclitaxel treatment for A, E. no treatment control; B, F. 6 hour treatment; C, G. 12 hour treatment; D, H. 24 hour treatment The first row features dot plots of mitochrondrial potential depolarization and caspase activation, detected by Mitotracker Red and Cell Event Caspase 3/7 Green reagents, respectively. The second row displays dot plots of phosphatidylserine exposition and complete compromise of cell plasma and nuclear membranes detected by a viability dye. **I.** Graphical representation of the percentages of cell death stages based on combinations of markers in the flow cytometric analysis. * (p<0.05), ** (p<0.01), *** (p<0.001). **J.** A linear correlation between the designated mitotic catastrophe index and midband fit at 25MHz from indicated time points and control. Curved lines indicate 95% confidence bands of regression lines. The goodness of fit was r2 = 0.991.

In order to investigate the statistical correlation between ultrasound parameters and the stage of cell death, cells were labelled with stains marking for mitochondrial depolarization (induction phase), caspase activation (initiation phase), phosphatidylserine detection (early execution phase), and viability compromise (late execution phase). It was observed that whereas mitochondrial depolarization and caspase activation occurred in the established chronological order (Figure 5A-5D), phosphatidylserine detection and viability compromise appeared to occur within a relatively short time frame, as indicated by the lack of cells staining positive for phosphatidylserine while negative for the viability compromise (Figure 5E-5H).

Based on these stains, it was observed that within a 24-hour period, the majority of dying cells appeared to be within either a cell-death induction or initiation phase (Figure 5I). The percentage of cells with initiation phase was selected as an index for cell death, as several of the morphological changes observed are the result of the action of caspases and their effector enzymes. Plotting this index against midband fit at 25 MHz showed a high level of correlation (Pearson coefficient of 0.9954; p<0.01) (Figure 5J). The goodness of fit of the linear regression was r^2^ = 0.991, suggesting that midband fit may be an accurate parameter to measure in estimating the death index of a tumour population. Spectral intercept, a related acoustic parameter to the midband fit also displayed a strong correlation with the cell death index based off the 25 MHz measurements, with a Pearson coefficient of 0.9767, a statistical significance of p<0.05, and the goodness of fit of the linear regression being r^2^ = 0.9539.

## DISCUSSION

This study demonstrated the use of high-frequency quantitative-ultrasound spectral radiofrequency analysis to detect structural changes in cancer cells in response to chemotherapy. The work confirmed the discriminatory ability of HFUS in detecting the predominant mode of cell death is not limited to radiotherapy, and correlated the cell death index with acoustic parameters. Based on the findings here, the trends in acoustic parameters can stem from alterations in the structure and organization of cell aggregations, as those found in a tumour.

Cell death effects have been previously demonstrated to be consistent using a variety of cell types *in vitro* and tumour types *in vivo.* Previous analyses of different cell lines *in vitro* and *in vivo* using radiofrequency analysis of HFUS data, include epithelial [20], AML-3 [18], PC3 [21], and MDA-MB-231 cells under different therapeutic regimens [10] in addition to bladder, head and neck, melanoma, breast and prostate tumour xenografts. The primary focus of this study was to characterize the capacity of HFUS to detect cell death over a short time period (≤12 hours). A 1 μM paclitaxel concentration was necessary to induce a sufficient amount of cell death of MDA-MB-231 cells for such a time period and hence was used as a death induction mechanism for this work. In selecting a different cell line and drug inducing mitotic arrest followed by cell death, previous investigations [18, 27] and experiments in this study had already determined that 0.25 μM colchicine (0.1 μg/mL) is sufficient to induce mitotic arrest in a significant number of AML5 cells.

Centrifuged cell samples have been employed in this study as a simplified *in-vitro* model to examine the interaction between spectral ultrasound parameters and changes in the cells due to biological processes following mitotic arrest. This allows for the correlation between specific cellular changes at different time points during treatment and associated changes in ultrasonic parameters. This eliminates complexities introduced in more complex tissues, including extracellular matrix and structure and blood flow. Previous studies have shown equivalent packing between dead and viable cells as prepared here, demonstrating intracellular features to be the principal cause of the differential ultrasound backscatter observed [22].

Ultrasound frequencies in this study are higher than those used in most clinical devices. However, there are numerous applications where high-frequency ultrasound is being used or developed for specifically clinical applications, including skin imaging [23], eye imaging [24], and catheter based techniques such as intravascular ultrasound and endoscopic based techniques [25]. Previous studies have shown the ability to detect changes as cells undergo apoptosis with high-frequency ultrasound and more recent work has shown the ability to detect apoptosis in clinical tumours with clinical ultrasound frequencies [10]. These results offer the potential to translate these results to clinical frequencies.

As observed in electron microscopy, paclitaxel treatment induced the formation of multiple large, highly- condensed scattering structures (~1-2 μm diameter). This supports the finding of increased attenuation [26], as larger, physically-dense scatterers will increase the degree of scattering in a sample, which can contribute to the attenuation of acoustic energy.

Previous studies have also estimated the spectral slope and midband fit in the characterization of diseased or tissue exposed to a variety of therapeutic agents [27, 28, 29]. The model proposed by Lizzi *et al.* [30] concludes that, assuming a random distribution of scatterers, spectral slope is inversely proportional to scatterer size once normalized power spectra have been corrected for attenuation. In a previous study by Kolios *et al.* [27], the spectral slope increased in cell samples undergoing classical apoptosis, a mechanism of death that features cellular shrinkage and loss and fragmentation of organelles into smaller fractions [31]. In this mode of cell death, the decrease in size of possible candidates for major scatterers, such as cell organelles or condensed chromatin around the nuclear envelope [32], corresponded well with the predicted decrease in scatterer size.

Within the present study, it was determined that the spectral slope decreased as MDA-MB-231 and AML5 cell populations were undergoing a form of cell death following mitotic arrest. Electron microscopy confirmed some characteristics of cell death mechanisms following mitotic arrest, with patches of condensed chromatin surrounding the nuclear periphery [33] and eventual formation of membrane-lacking, highly-condensed chromatin bodies in the cytoplasm of cells [36]. Additionally, as with several other studies [34, 35], TUNEL-negative staining was observed for cell populations undergoing certain forms of cell death post-mitotic arrest. The lack of positive phosphatidylserine staining while staining negative for viability compromise is another supportive observation. A study by Morse *et al.* [36] involving docetaxol, a related taxane to paclitaxel, in the treatment of MDA-MB-231 cells had also observed minimal (<1%) increases in Annexin-V positive, propidium iodide negative staining. Evidently, the predominant form of cell death by microtubule inhibitors such as paclitaxel or colchicine is initiated after mitotic arrest [37], leading to a larger collection of cells to be at the G2/M interface. As stated previously, scattering theory predicts that spectral slopes are smaller for cells containing larger scatterer size. As cells and nuclei within G2/M are known to be considerably larger than cells and nuclei within G1/G0 [38, 39], and hence it is likely that the observed decrease in spectral slope is a reflection of the increase in number/percentage G2/M cells undergoing cell death. This is further supported by a correlation between the percentage of G2/M cells and spectral slope. Therefore, the observed decreases in spectral slope and increases in estimated scatterer diameter provide a benchmark for using high-frequency ultrasound to differentiate the prevailing manner of cell death in not only radiotherapy, but also chemotherapy.

It should be noted that although the ESD trends likewise suggested an increase in scatterer size, the cause of the disparity between 25MHz and 40MHz ESD values is most likely related to the fact that the two different ultrasound frequencies are primarily interrogating different cellular structures. Studies by Oelze *et al*. [40] have demonstrated that differences may arise for such parameters depending on the frequency bands analyzed. In this case the difference in values stem from the difference in using analysis bandwidths of 13-32MHz and 22-52MHz for the 25MHz and 40MHz measurements, respectively.

Multiple sample parameters, including the concentration, compressibility, and spatial arrangement of acoustic scatterers [5, 7, 41] can affect ultrasound backscatter. Additionally, the speed of sound may also influence the backscatter data [7, 30, 42]. However, within this study, the small variance and consistency of this the speed of sound with previous tissue characterization [43] diminishes the possibility of it severely affecting results. Increases in midband fit at the transducer's central frequency have been found to be a common marker in all studied cases of samples exposed to chemotherapy, photodynamic therapy, and radiotherapy [15, 41, 44]. Current studies seem to suggest that this increase has a strong connection with the status and arrangement of nuclear material within treated cells [27, 41]. According to theoretical models, increases in the number of dense scatterers would amount to increases in the backscatter, reflected as increases in the spectral intercept and midband fit Electron microscopy demonstrated the formation of highly condensed conglomerations of nuclear material. Given the size of these aggregates, it is possible that they may act as individual scatterers, thereby increasing the scatterer concentration. This is supported by output from the Fluid-Filled Sphere model in this study, suggesting that the estimated effective acoustic concentration increased as a function of paclitaxel treatment. Previous studies have also noted that the addition of DNAse to treated samples containing condensed nuclear material resulted in decreases in backscatter [18].

The current working model of ultrasound backscatter in the context of cell death hypothesizes that structural changes around the nucleus and nuclear material contribute significantly to an increase in backscatter. Multiple lines of evidence exist to support this. Firstly, the acoustic nuclear signal may be used to differentiate tumor cell lines of different origins [47]. Colchicine induction of heterochromatin resulted in significant increases in the ultrasound backscatter, which in turn was reversed by subsequently administering DNase to decrease nuclear density [27]. Treating solely with DNase resulted in backscatter signals decreasing by at least 50 percent relative to pretreatment values [45]. Comparably, inducing chromatin unfolding by treatment with sodium butyrate also resulted in significant backscatter decrease [45]. In addition, in experiments isolating nuclei from apoptotic versus viable cells, apoptotic nuclei displayed significantly greater backscatter [15]. Lastly, the backscatter spectra of xenograft tumor and centrifuged cell samples demonstrated remarkable similarities despite the latter system lacking vasculature and an extracellular matrix [16].

Based on the working hypothesis that condensed nuclear material is predominantly responsible for the increases in backscatter, the cell death index was chosen to incorporate cells that had activated caspases following mitochondrial depolarization but had not progressed as far as compromising membrane integrity. It was reasoned that such a cell population would have progressed far enough into the cell death programme as to contain the observed chromatin aggregates resulting from the actions of activated caspases, but not progress so far as to have nuclear material escape due to a porous plasma membrane – as observed in the ISEL staining at 24 hours. Cells undergoing primary necrosis, with or without caspase activation, were also not a part of the cell death index. The finding that midband fit correlated most strongly with this particular cell population provides two important conclusions. The first is that this observation provides further support for the general hypothesis of condensed nuclear material being the major scattering source in the course of chemotherapeutic treatment by paclitaxel. The second is that changes in acoustic parameters possess a quantitative relationship with the degree of cell death induced by paclitaxel. Therefore, it is possible that the midband fit and related acoustic parameters may be used as biophysical markers to quantitatively assess the degree of cancer cell death in cell samples exposed to therapeutic effectors.

## IMPLICATIONS

The malignant breast cancer line MDA-MB-231 and acute myeloid leukemia line OCI-AML5 were selected for this study due their derivation from a chemotherapy treated cancer type [46]. Therefore, for both cell lines it becomes increasingly important for differentiating the mode of cell death induced by the intended drug from other background cellular death processes so as to eliminate the chance of a false positive conclusion on therapeutic efficacy. Additionally, an accurate estimation of cell death index through a rapid, non-invasive, and inexpensive technique would allow for very effective monitoring tumour response and, if necessary, adapting treatment when needed.

## CONCLUSION

High-frequency ultrasound (>20MHz) quantitative radiofrequency analysis methods were used to detect response to chemotherapy treatment *in vitro*, confirm the potential for ultrasound spectral slope as a parameter capable of differentiating the primary mode of cell death in chemotherapy, and effectively establish a correlation between cell death index and acoustic parameters of tumour-mimicking systems. These results provide a framework for future experiments with the goal of establishing a set of benchmarks for the accurate estimation of cell death and therapeutic efficacy through an ultrasound-based approach. This provides incentive for the further characterization of tumour response in preclinical animal models as well as for further research into the establishment of acoustic parameters as markers for quantifying the degree of cancer cell death, and by extension, the efficacy of treatment.

## MATERIALS AND METHODS

### Cell culture

MDA-MB-231 cells (ATCC, Manassas, VA), obtained from frozen stock samples, were cultured in RPMI-1640 media (Wisent, Montreal, QC) supplemented with 10% fetal bovine serum and 1% Penicillin- Streptomycin and incubated 37°C and 5% CO_2_.

OCI-AML5 cells were derived from a leukemia patient and kindly provided by Dr. Minden (Princess Margaret Cancer Centre, Toronto, ON) were cultured in AMEM media (Wisent, Montreal, QC) supplemented with 5% fetal bovine serum and 1% Penicillin-Streptom and incubated 37°C and 5% CO_2_.

Cells were maintained in an exponential growth phase, and cultured to appropriately sized populations as required by the experiment.

### Determination of paclitaxel concentration (supplementary)

Treatments to induce cell death were carried out by dosing cells at 80% confluence with final concentrations of 0.01 μM, 0.1 μM, and 1μM of paclitaxel (Bristol- Myers, Montreal, QC) added to the growth medium. Cells were returned to 37°C, 5% CO_2_ growing conditions for 24 hours, and then the non-adherent and adherent cells were isolated and counted by haemocytometer. The population of adherent cells as a percentage of total cell population was used as an estimate for selection of drug concentration that would produce a substantial (>20%) amount of non-viable cells. Based on haemocytometer readings on the percentage of floating cells relative to the total cell population (Figure S3), an end concentration of 1 μM paclitaxel was chosen for all treatment conditions. Control samples were time matched and received no drug. The colchicine dosage for AML cells was pre-determined based on previous ultrasound characterization studies [18, 27] and was 0.1 μg/mL.

### Cell sample formation

Following the selection of a paclitaxel concentration to use, treatment time points were chosen to be 6, 12, and 24 hours of paclitaxel exposure, and time-matched untreated control. Treatment was administered to cell populations in T125 flasks at 80% confluence. For each experimental time point following paclitaxel treatment or control, 5.0 × 10^6^ cells were trypsinized and transferred to 50mL centrifuge tubes, followed by centrifugation at 240g for 5 minutes. Afterwards, media was aspirated and cells were resuspended with 200μL of phosphate buffered saline (PBS) with present divalent cations. 100μL of this suspension (2.5 × 10^6^ cells) was then transferred to one of the wells of the custom three-welled chamber. A second round of centrifugation at 1500 g for μminutes produced the desired packed cell samples of approximate 1 mm height and 8 mm diameter. The sample-containing chambers were then immersed in a solution of PBS with present divalent cations for subsequent ultrasound imaging. The remaining 100μL of the cell suspension was used to create a parallel sample for histological analysis. This was carried out as previously [47].

### Ultrasound imaging

Cell samples were imaged in two separate wells of a custom 3-well sample holder, with the third well used as a calibration reference required for obtaining the necessary ultrasound parameters, as described in Taggart *et al* [21]. Each cylindrical well was 8 mm in diameter and 3 mm deep. The bottom of each well was polished steel to act as a planar reflector for the ultrasound. The entire sample holder fit into a custom centrifuge holder for the second round of centrifugation to form samples for ultrasound analysis. All measurements took place at room temperature using a Vevo770 (VisualSonics Inc., Toronto, Canada) high-frequency ultrasound device using 25 MHz and 40 MHz centred transducers with f-numbers of 2.1 and 2.0, respectively, as well as analysis bandwidths of 13-32MHz and 22-52MHz, respectively. Six planes of raw radio- frequency data were acquired from each of the samples and the corresponding PBS only well. All ultrasound parameters were derived from radiofrequency data collected based on previously-established methodologies [14, 47].

The acoustic attenuation was estimated using an insertion loss method, subtracting the power spectrum from the planar reflector at the back of a reference well from the planar reflector beneath the sample, to give the frequency dependent attenuation [14]. The speed of sound was estimated using the reference well as a known depth relative to the sample wells. Additional quantitative parameters were calculated for regions 15 by 15 wavelengths in size tiled across sample data. Within each region the power spectra were calculated and normalized by the reference power spectrum. A line was fit to the normalized power spectrum over the transducer bandwidth and the spectral slope and spectral intercept were determined along with midband fit (intensity of fitted line at the centre of the frequency band) [17]. From the normalized power spectrum, the backscatter coefficient (BSC), a fundamental material characteristic of the sample describing the echogeneity, was estimated based on the method established by Chen *et al.* [48].

From Insana and Hall [7] Eq. 4 the BSC can be described as:

$$\text{BSC}\left(f\right)={\text{Cf}}^{4}D^{6}\bar{n}\gamma^{2}F\left(f,D\right),$$

where *C* is the constant *π*^4^/36*c*^4^ with *c* the speed of sound in the medium. *F(f, D)* is the form factor which describes the change in shape of the BSC as a function of frequency (*f*) and the scatter diameter (*D)*. The effective acoustic concentration (EAC) is the combination of the volumetric number density ($\bar{n}$) and the relative impedance mismatch between the scatterer and surround medium (*γ*) squared, $\bar{n}\gamma^{2}$. In order to obtain an estimate of the effective scatterer diameter (ESD) for the samples, the form factor for a fluid filled sphere was used as a model (13). The form factor for the fluid filled sphere is expressed as:

$$\text{BSC}\propto {\text{f}}^{4}\left(\frac{3}{\text{kD}}\right)j_{1}{\left(\text{kD}\right)}^{2};$$

where f is the frequency, k is the wavenumber, D is the scatterer diamete]r being estimated as our ESD and j_1_ is a spherical Bessel function of the first kind and first order. For each region, the fluid filled sphere model of the BSC was fitted to the data in order to estimate the ESD and EAC [12].

### Histology

Packed cell samples were fixed using 10% (w/v) formalin (Fischer Scientific, Mississauga, ON) for 48 hours at 4°C and subsequently embedded in 3% agarose and processed into paraffin sections and slides. Parallel samples were used for haematoxylin and eosin (H&E) and ISEL staining to observe cellular morphological alterations and DNA fragmentation. H&E Staining was done according to standard staining protocol [49], and ISEL staining followed the protocol of Wijsman *et al*. [50] using the *In Situ* Apoptosis Detection kit (R&D Systems, Minneapolis, MN), following manufacturer's instructions. Slides were sealed with Cytoseal (Fischer Scientific Mississauga, ON) and imaged within 3 weeks of preparation.

### Electron microscopy

Packed cell samples were fixed in 2.5% (w/v) glutaraldehyde (Fischer Scientific, Mississauga, ON) with 0.1M sodium cacodylate buffer (Electron Microscopy Sciences, Hatfield, PA) for 48 hours at 4°C, stained with 1% osmium tetroxide, and dehydrated [47] Samples were then polymerized and imaged. Imaging was carried out using an electron microscope (Manufacturer and Model, City) operating at 80 keV energy and at 10000x magnification.

### Cell cycle analysis

For each of the treatment times or time-matched control, cells were dissociated from their flasks by trypsin and then fixed in 4% (w/v) paraformaldehyde (Fischer Scientific, Mississauga, ON) for 60 minutes at 4°C. Cells were permeablized by 0.2% (w/v) Triton-X100 (Sigma Aldrich, St. Louis, MO) for 5 minutes at room temperature, and then incubated with propidium iodide/RNase A stain (Molecular Probes, Eugene, OR) for 30 minutes at 37°C in the dark.

Flow cytometry measurements were performed using a BD LSRII flow cytometer (BD Sciences, San Jose, CA), with 488nm light exciting PI to emit at a wavelength of 610nm, captured through the TexasRed bandpass filter. Cell cycle analysis was performed using FCS Express 4 Multicycle software (De Novo Software, Glendale, CA).

### TUNEL assay

A TUNEL assay (Roche Diagnostics, Mississauga, ON) was performed according to methods previously described [51] Before FITC-TUNEL reagent addition, six samples of 5×10^6^ cells were prepared. They were 1) untreated MDA-MB-231 cells; 2) MDA cells treated with DNAse (3000U/mL; GenScript, Piscataway, NJ); 3) 24- hour paclitaxel-treated MDA cells; 4) a 1:1 mixture of untreated and DNAse treated cells; 5) a 1:1 mixture of paclitaxel and DNAse treated cells; and 6) a 1:1 mixture of paclitaxel treated and untreated MDA cells.

Flow cytometry measurement was performed using a BD LSRII flow cytometer, with 488nm light exciting the fluorophore to emit at a wavelength of 530nm, captured through a FITC bandpass filter. Further analysis for TUNEL stained samples was carried out using FlowJo v7.6.5 software (FlowJo, Ashland, OR).

### Cell death analysis

Four-colour flow cytometry was used to quantify cell death. Cells were either untreated, or exposed to 1 μM paclitaxel for 6, 12 or 24 hours, then dissociated, and stained with primary mouse IgG anti-phosphatidylserine antibody (2μg/mL; Millipore, Etobicoke, ON); MitoTracker Red (50nM; Invitrogen, Burlington, ON); LIVE/DEAD Far Red Dead Cell (1:500 dilution; Invitrogen, Burlington, ON); and Cell Event Caspase 3/7 Green Detection Reagent (5μM; Invitrogen, Burlington, ON) with a secondary antibody of goat anti-mouse IgG antibody conjugated to AlexaFluor405 fluorophore (5 μg/mL; Invitrogen, Burlington, ON). Approximately 10000 events were measured for each time point using a BD LSRII flow cytometer and analyzed using third party program FCS Express 4 (De Novo Software, Glendale, CA).

### Statistical tests

For all variables, one-way ANOVA was performed comparing time points. Where statistically significant differences were detected, post-hoc Tukey tests were carried out for each experimental time relative to the control sample to ascertain statistically significance. Pearson's correlation coefficient was used to assess the relationship between two continuous variables (i.e. estimated scatterer diameter against the percentage of cells in G2/M). Analyses were performed using the statistical analysis program GraphPad InStat 3.1 (GraphPad 2003, La Jolla, CA).

Statistically significant differences were considered for p<0.05 (indicated by *), p<0.01 (**), and p<0.001 (***). Non-significant differences (NS) between the time points relative to the control were indicated.

## SUPPLEMENTARY FIGURES


