# Supplementary material for: High-frequency ultrasound analysis of post-mitotic arrest cell death
Source: Oncoscience. 2016 Apr 15;3(3-4):109–21. doi: 10.18632/oncoscience.301 (PMC4872649; doi:10.18632/oncoscience.301)
Supplement: Supplementary file 1 [file oncoscience-03-109-s001.pdf]

SUPPLEMENTARY FIGURES

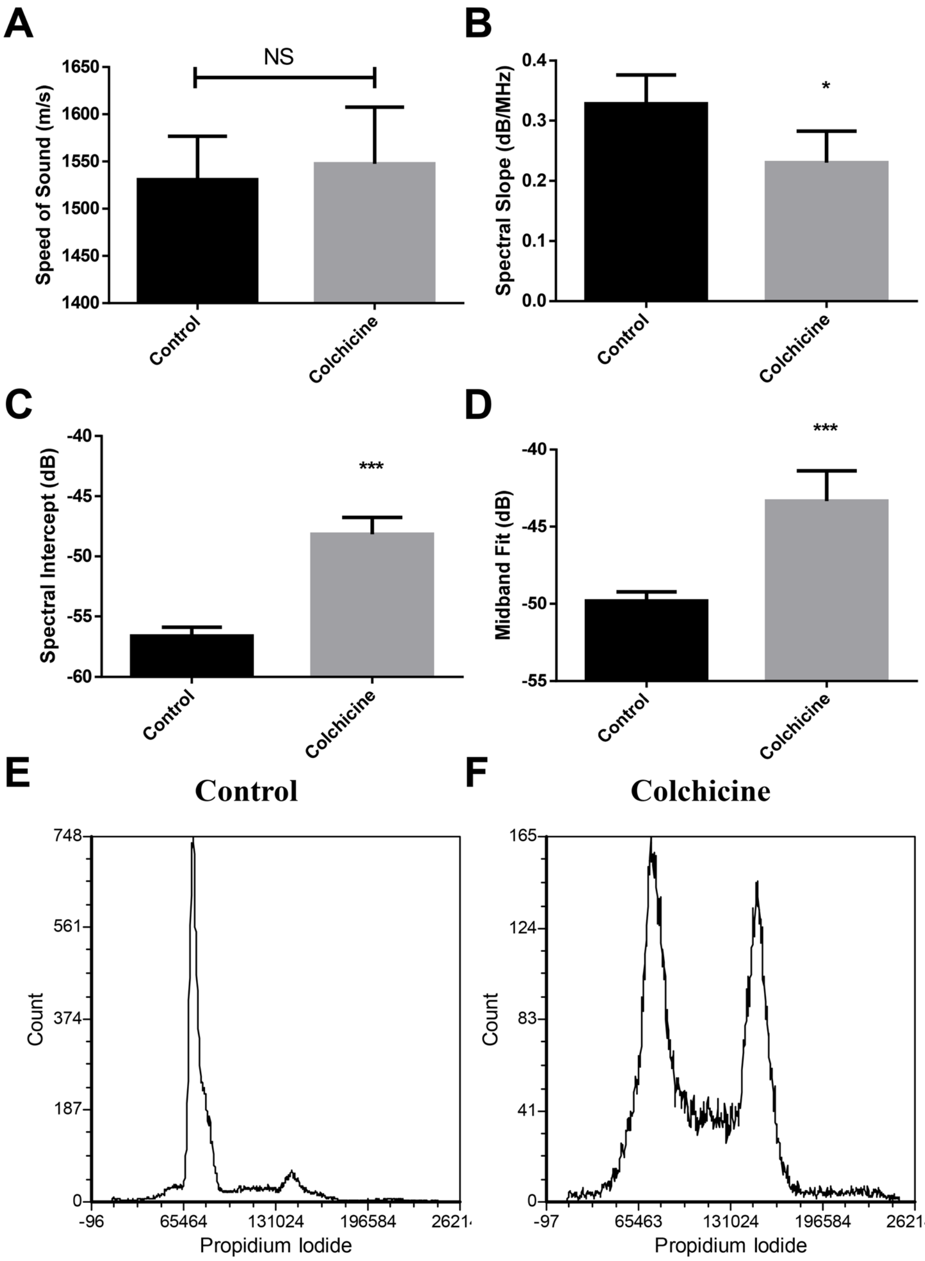

Supplementary Figure S1: Spectral ultrasound and cell cycle changes from 24-hour colchicine-induced mitotic arrest in AML5 cells. Ultrasound parameters **A.** speed of sound, **B.** spectral slope, **C.** spectral intercept, and **D.** midband fit demonstrated similar trends to those observed for MDA-MB-231 cells. Measurements were performed using a transducer with a 20 MHz central frequency. Cell cycle profiles for **E.** untreated control and **F.** 24-hour colchicine observably demonstrated in increase in G2/M content populations, indicative of mitotic arrest. Error bars represent standard deviation. n ≥ 3 for all conditions. NS (not significantly different), * ($p<0.05$), *** ($p<0.001$).

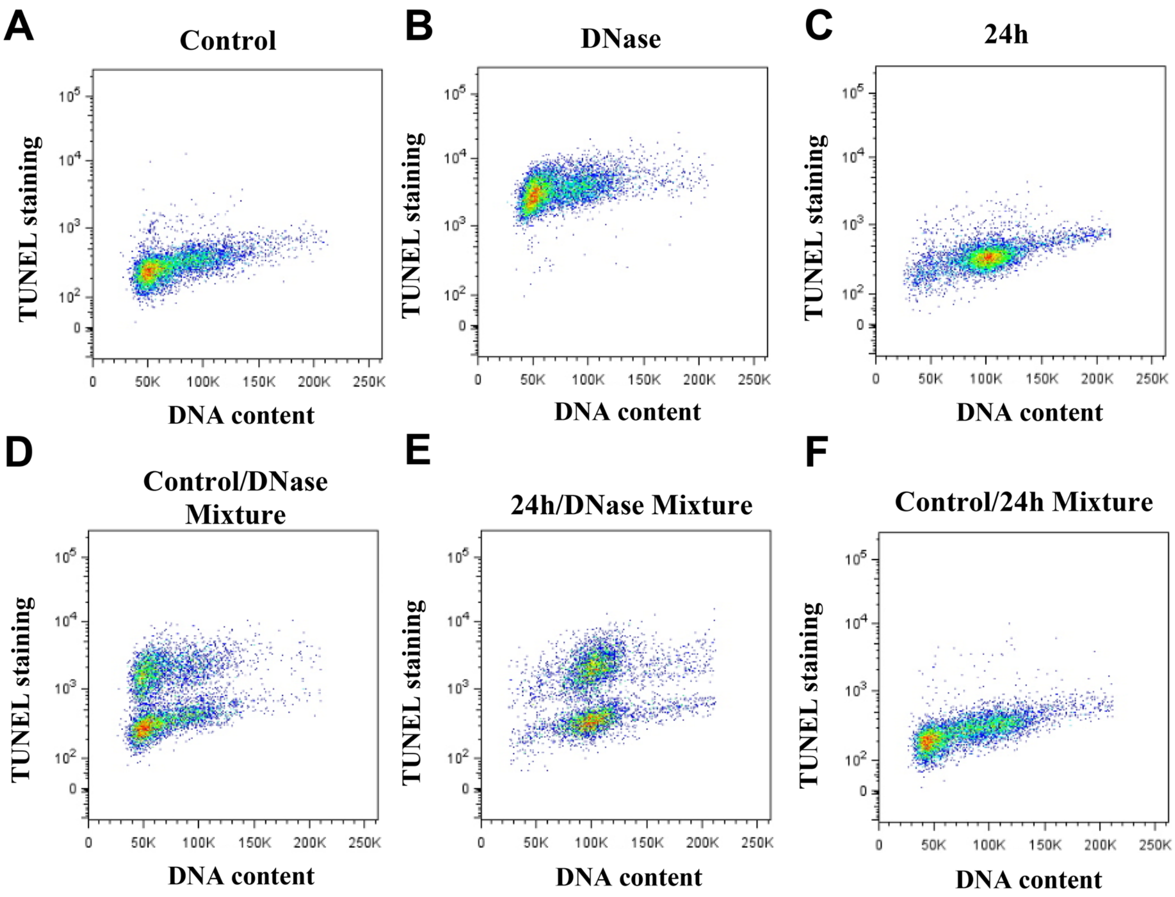

Supplementary Figure S2: Flow cytometric analysis of TUNEL staining. **A.** untreated MDA-MD-231 cells; **B.** MDA cells treated with DNAse; **C.** 24-hour paclitaxel-treated MDA cells; **D.** a 1:1 mixture of untreated and DNAse treated cells; **E.** a 1:1 mixture of paclitaxel and DNAse treated cells; and **F.** a 1:1 mixture of paclitaxel treated and untreated MDA cells.

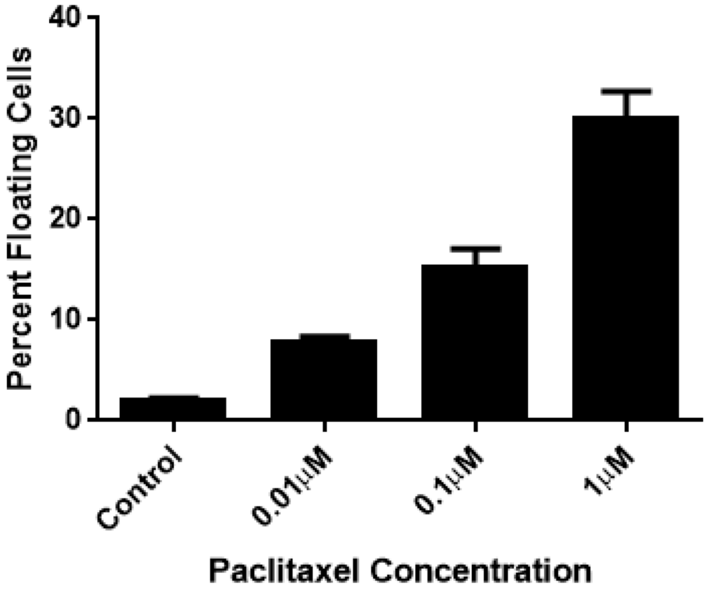

Supplementary Figure S3: Determination of a paclitaxel concentration to induce appropriate amounts of cell death after 24 hours. Floating cells were presumed to be non-viable and taken to represent the expected minimum number of cells undergoing cell death in subsequent flow cytometry death analysis. Bars represent standard deviation. n=4 per condition.
